# On the “Duel” Nature of History: Revisiting Contingency versus Determinism

**DOI:** 10.1371/journal.pbio.1000259

**Published:** 2009-12-15

**Authors:** Brian Andrew Swartz

**Affiliations:** 1 Department of Integrative Biology, University of California Berkeley, Berkeley, California, United States of America; 2 Museum of Paleontology, University of California Berkeley, Berkeley, California, United States of America

## Abstract

Are we “historical accidents” of an undirected evolutionary history? In his recent book, *Islands in the Cosmos*, Dale Russell addresses this question, and Brian Swartz reviews his synthesis of this “cosmic” evolutionary debate.

*“Organisms are not billiard balls, propelled by simple and measurable external forces to predictable new positions on life's pool table. Sufficiently complex systems have greater richness. Organisms have a history that constrains their future in myriad, subtle ways.”—Stephen Jay Gould (1980: 16)* [1]

What is the relationship between external—physical and biological—influences on increasingly complex matter over billions of years? In his most recent book, *Islands in the Cosmos: the Evolution of Life on Land*, Dale Russell attempts to answer this question. Russell is the senior curator of paleontology at the North Carolina Museum of Natural Sciences and, among other things, is well known for proposing in 1971 an extraterrestrial cause for the Cretaceous-Tertiary extinction that wiped out the non-avian dinosaurs [2]. This places Russell among the first paleontologists to consider this extinction as a relatively sudden event in Earth's history. Dale Russell has spent his lifetime pondering grand evolutionary questions, and it is a quest that *Islands in the Cosmos* well illustrates.

His central thesis explains that evolutionary theory (as he views it)—based on “random mutations” and “adaptation to irregular changes in the physical environment”—inadequately accounts for long-term trends in the competitive abilities of organisms and the ultimate appearance of sentient beings (e.g., *Homo sapiens*) in the cosmos. Russell instead proposes that even though mutations are random, because “…the effects of natural selection are not random, and modulated by adaptive responses to irregular changes in the physical environment,” properties of matter and feedback in biotic competition have established a deterministic trajectory in the history of life. His argument distills to the following interrelated points:

- The universe is fine-tuned for life as we know it.
- The physical workings of the Earth (e.g., its radius and tilt, mantle convection, the heterogeneity of continental surfaces, and a long-term stable environment) favor the origin of life and evolution of multicellular terrestrial beings.
- Positive feedback and competitive interactions among organisms increase evolutionary rates, competitive abilities, activity levels, and behavioral complexity of these beings over time.
- Convergent evolution is a testament to these precepts, and sentient beings are a natural and emergent outcome of these processes.

Although he broadly frames these issues in the introductory pages and synthesizes them in the final chapter, Russell dedicates the bulk of this self-proclaimed essay to a chronology of evolution in the broad sense, as “change through time”. In 289 pages (82% of the book), Russell retells the 13.7 Ga (billion year) history of our cosmos and the ∼3.8–3.5 Ga history of life on Earth. He provides occasional “hints” of his thesis in the larger chapter text and briefly elaborates on them in the final paragraphs of each chapter, but for the most part, his main ideas seem to get lost in the bombardment of historical and paleoecological facts through time. His love of dinosaurs and of the Cretaceous Period certainly shines through the wash of information. For example, when framing the seven most important scientific debates over the last 50 years, three of the seven questions include controversy over dinosaurian evolution. Unfortunately, however, in an attempt to pull information from such a broad range of disciplines, there are several mistakes and irregularities that the specialist might find distracting. For example, instead of explaining that structures and sequences (as opposed to whole organisms) are recapitulated during ontogeny [3], Russell incorrectly argues that we replay “the previous evolutionary history [of our] ancestors” (pg. 120).

Russell's synopsis of the cosmos, the Earth, and life through time might otherwise interest the triviaphile, but it is difficult to know his intended audience. Specialized terms are often used without explanation (e.g., Opiliones, an obscure group of arachnids), and basic concepts are commonly followed with reasonable elaboration (e.g., natural selection). Irrespective of his intent to commit much of his essay to the historical record (i.e., so that if rejected, his thesis “…will not detract from an appreciation of the history of terrestrial life on our planet…” [pg. 319]), his ideas nonetheless touch upon several important evolutionary questions.

Russell's central question is whether evolution is “random (contingent) or directional (teleological)” (pg. 7). Although he passionately argues for ultimate direction (i.e., determinism) and promotes the view of being “inevitably human in a lonely universe” [4], he never fully explains what contingency means (it is simply when preexisting events determine the nature of succeeding ones), or relates his thesis to essential evolutionary notions that naturally emerge from these “ultimate” evolutionary questions. Thus, to understand his thesis more fully, it is necessary to explain a few additional evolutionary details.

Russell's major biological precepts derive from the modern synthesis—the intellectual fusion of Darwin's concept of natural selection with Mendel's “heritable factors,” or genes [5]—and he clearly subscribes to the view that variation is copious and non-directional, that natural selection as an external driver acts at the level of organisms to impose direction, that organismal competition promotes divergence (like Darwin's “principle of divergence” [6]), and that the full panoply of life's diversity arises when these tenets are extended (i.e., extrapolated) over geologic time [5]. Although his addition to these precepts includes the argument that physical and biological influences impose ultimate direction on life's trajectory, his thesis is fundamentally founded in the dogma of the modern synthesis. Thus, by ignoring and/or rejecting the historiographic account of evolutionary ideas over the last 150 years [7], Russell has come to argue that macroevolutionary direction derives from the role of external influences and extrapolationist thinking. In other words, in his view, external factors impose a deterministic outcome on the history of life.

In following, Russell's argument for the overarching roles of the “external” and of the “deterministic” quickly break down when the subsequent evolutionary data, which he does not reflect upon, are considered: (a) internal processes like development and genealogical history constrain (or channel, as a positive definition) variation and evolutionary direction over time [7]–[10], (b) the intersection of this “channeled” variation is random relative to the specific environment a lineage inhabits over geologic time [11], and (c) there are certain characteristics that individual organisms cannot possess—like the tendency to vary or a geographic range—and which instead characterize lineages. This emerges over macroevolutionary time in processes like sorting and clade selection, whereby natural selection acts on lineages to produce trends that do not merely result from competitive interactions among individuals [12]–[17].

These data unavoidably compromise Russell's thesis, as the argument now shifts to the role of internal and emergent (i.e., non-extrapolationist) processes laying their contingent fingerprints on life's trajectory. However, there is no need to create false dichotomies. There appears good evidence that variation is channeled by internal and external sources [18],[19], that evolution is hierarchical and selection acts at multiple levels, and that some—but certainly not all—microevolutionary processes extrapolate over geological time [20]. Furthermore, whereas ecological determinism (determinism in the small sense, over short time scales) may set limits on the range of feasible adaptations to a particular environment [21], contingency unavoidably acts at every biological tier and thus naturally imparts direction from chance interactions over all facets of evolutionary time.

*Islands in the Cosmos* is another attempt in recent literature [4],[22] to hypothesize determinism as an ultimate outcome in the evolution of life. However, contrary to the dichotomy Russell proposes, this view should not be seen as an alternative to contingency. Although famous authors (e.g., GeeratVermeij) and great papers [23] may draw upon evidence of convergent and parallel evolution to argue for ecological determinism, these arguments are not at odds with the role of contingency—whether in exploitation and opportunism, or as a major player in the assemblage of biochemical pathways, morphological features, or diversity patterns—over evolutionary time.[Fig pbio-1000259-g001]

**Figure pbio-1000259-g001:**
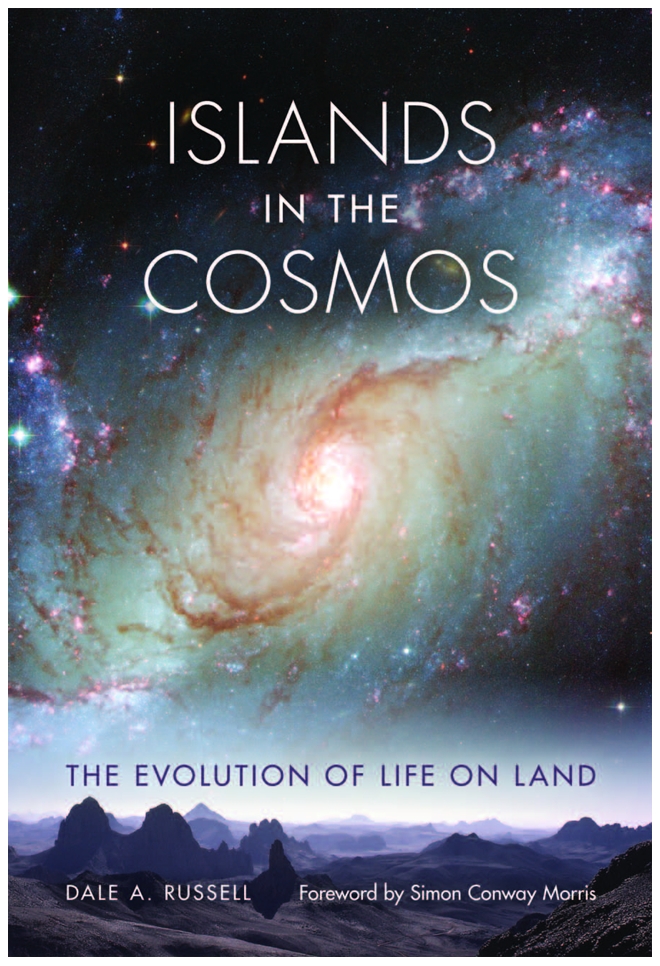
Russell, Dale A (2009) Islands in the cosmos: the evolution of life on land. Bloomington IN: Indiana University Press. 480 p. ISBN (hardcover): 978-0-253-35273-6 US$34.95.
